# Interdisciplinary approach to defining outdoor places of knowledge work: quantified photo analysis

**DOI:** 10.3389/fpsyg.2023.1237069

**Published:** 2023-12-01

**Authors:** Aulikki Herneoja, Emilia Rönkkö, Annu Haapakangas, Sara Malve-Ahlroth, Essi Oikarinen, Simo Hosio

**Affiliations:** ^1^Faculty of Technology, Oulu School of Architecture, University of Oulu, Oulu, Finland; ^2^Finnish Institute of Occupational Health, Helsinki, Finland; ^3^Center for Ubiquitous Computing, Faculty of Information Technology and Electrical Engineering, University of Oulu, Oulu, Finland; ^4^Tokyo College, University of Tokyo, Tokyo, Japan

**Keywords:** outdoor knowledge work, outdoor reading, outdoor and semi-outdoor environments, built environment, physical work environment, interdisciplinary approach, photo analysis, machine-vision analysis

## Abstract

**Introduction:**

Working outdoors is an emerging, sparsely studied phenomenon in knowledge work. Office tasks have traditionally been considered to belong to indoor environments. The worldwide pandemic of COVID-19 has increased and changed attitudes toward multilocational working. The aim of this method study is 2-fold: first to define for interdisciplinary context outdoor environments when they are used as places of outdoor knowledge work and second to quantify the thematic photo analysis to support interdisciplinary understanding of the places of outdoor knowledge work.

**Methods:**

The review of literature has been one of the methods to support the interdisciplinary approach of this article. The photographs of outdoor knowledge workplaces and views from the workstations are studied through photo analysis customized from the existing press photograph analysis.

**Results:**

First, we defined outdoor environments when used as places of outdoor knowledge work, as unconditioned outdoor or semi-outdoor places (opposite to closed indoor spaces with stable, conditioned indoor climate) providing favorable action possibilities as sources of comfort and mitigating unfavorable conditions, for example, by microclimatic solutions. Instead of defining all spaces as outdoor environments not fulfilling a stable indoor climate (conditioned) definition, adaptation to thermal and physical environments also brought semi-outdoor space into the definition. In this context, favorable latent action possibilities (affordance) in the built environment are often related to microclimate as a source of comfort. Second, we focussed on photo analysis. The proposed model is based on journalistic photo analysis PPSA and the pOKW model, which have been further developed in this study to pOKW2 model for analyzing mobile-based collected self-reported photographs by the occupants. In this pOKW2 model, the photographs would have time-location information enabling the combining of data from other datasets and thereby reducing the number of characteristics to be analyzed from the photograph. We proposed rating (in numeric form) to detect the favorable and unfavorable characteristics in the photographs most likely supporting or hindering conditions of outdoor knowledge work. This quantification would enable the use of machine vision analysis and would support handling large quantities of photographs and their combination with other datasets in interdisciplinary research.

**Discussion:**

The quantification of the photo analysis (pOKW2) includes the readiness to combine the analysis results with other time-location-specific datasets in an interdisciplinary research collaboration to advance our understanding of latent action possibilities for outdoor knowledge work.

## 1 Introduction

Knowledge work has traditionally been considered to belong to indoor environments. In this context, working outdoors is still a novel phenomenon, and no established definition is available. Places of outdoor knowledge working have been examined at a general level, focussing on the location (requiring to be right outside the workplace), including characteristics of a green, lush, and tranquil enough environment, and the immediate surroundings of the worker (e.g., requiring for seating or safe and distant places from traffic and noise for “think walk” or “walk and talk”; e.g., Petersson Troije et al., 2021). However, the broader physical context of outdoor knowledge work and its possibilities in urban built environments have not yet received attention in this new area of research. In this study, we seek to widen the scope of research on outdoor knowledge work by applying methods and concepts of architectural and urban design research to grasp the qualities of the built and natural environments of outdoor working. As part of interdisciplinary research, qualitative photo analysis of places of outdoor knowledge work offers insights and sets demands for the broader multimethod approach.

The post-pandemic increase in working from home has generated new research on the physical hybrid work environments. Hybrid work means combining on-site working at the workplace and teleworking (e.g., at home office, cafes, coworking spaces), and the focus of research is mainly on indoor spaces. However, this has overshadowed the complexity of multilocational working and the larger ecosystem of physical places, including outdoor areas and transitions between locations. The increased spatial and temporal flexibility of working has profound and complex consequences on working and living arrangements and the qualities of the work environments that people are exposed to. Consequently, this transformation of the way of working should be reflected in the planning of workspaces, buildings, and urban areas. The boundaries of living and working environments are also blurred in the ecosystem of hybrid workplaces. In this context, working outdoors is an emerging, sparsely studied phenomenon, which seems to receive increasing attention in multimethod interdisciplinary research of wellbeing and health (e.g., Mangone et al., 2017; Gritzka et al., 2020; Rudokas et al., 2020; Hu et al., 2022).

There is no established definition of outdoor knowledge work in work environmental research. Traditionally, psychological research on physical work environments has conceptualized “work environment” and “workspace” as interior spaces of buildings in office work (e.g., Vischer, 2008; Spivack et al., 2009). Based on existing studies related to outdoor knowledge work, the concept may refer to various work activities (e.g., walking meetings and outdoor computer work), outdoor activities during breaks (breaks, transitions), and working in outdoor environments, such as outdoors of the workplace, home, and leisure home or in their immediate vicinity, or outdoor environments designed explicitly for outdoor working. Working outdoors has become technologically and socially possible due to increasingly flexible, autonomous, and multilocational working. There have been weak signals of its attractiveness and promotion even before (Telenor Sverige AB, August 9, 2016; The New York Times, January 1, 2019) and as a result of the coronavirus crisis (Dufva and Rowley, 2022). Societal megatrends such as the pandemic, the increase of knowledge work, interest in wellbeing, and the demands of sustainable development are creating pressure to transform the structures of working and living, including the physical work environment. Working outdoors itself has been studied in only a few studies (e.g., Plambech and Van Den Bosch, 2015; Petersson Troije et al., 2021), most of which only concern walking meetings (e.g., Bälter et al., 2018).

Hence, after discovering that there is no established definition for outdoor knowledge working or the spatial solution of an outdoor knowledge workplace, we proposed an approach (Herneoja et al., 2022), which we further advance in this article. We started with an aim to approach the prevailing situation by developing a visual analysis of the non-academic visual material published about design solutions, either designed for outdoor knowledge working or just used for outdoor office purposes. We had recognized that places and ways of outdoor work had raised interest in popular media as visual discovery engines such as Pinterest, where people share, in a non-professional and non-academic context, their images and ideas, or images from other media such as blogs, magazines, and suppliers' commercial webpages. We were aware of the possible commercial interest (Lo et al., 2016) and the use of algorithms based on artificial intelligence and machine learning with complex internal logic (Liu et al., 2017) on these visual discovery engines. Still, we were interested in exploring the places of outdoor knowledge work by analyzing the found images (Herneoja et al., 2022).

Photographic documentation and analysis have already been used to investigate the places and experiences of multilocational working by Florin and Lehtinen-Jacks (2022), but these photographs did not include time-location information. At present, the growing interest in interdisciplinary research of wellbeing and health, together with the possibilities of multimethod approaches for an in-depth analysis of context-sensitive time- and location-specific data on places (incl. affordances), behaviors, experience, and physiological responses, also challenges qualitative visual analysis methodologies. The mobile-based collecting of self-reported photographs of outdoor knowledge workplaces enables time-location-specific data to be included in the photograph. Time-location information enables combining other datasets in connection with the photographs, and not all information is needed to analyse from the photograph itself. This simplification would benefit the analysis of large quantities of photographs. In this study, the outdoor knowledge work purposes customized pOKW model (Herneoja et al., 2022) based on Kedra's (2013) PPSA is elaborated to pOKW2 model to improve its features when combining datasets in interdisciplinary research collaboration. In the pOKW2 model, we also considered the possibility of analyzing large quantities of photographs, where machine vision analysis would ease thematizing findings in quantified form.

## 2 Defining outdoor and semi-outdoor environments

In the building design framework, it is relevant to define the *outdoor environment* in relation to the indoor environment which is the typical place for knowledge work. In a technical context, the indoor environment is well-defined through the energy performance of buildings, i.e., indoor environmental quality (IEQ; BS ISO 17772-1, 2017). It seems relevant to assume that those indoor environments not fulfilling the set standards of IEQ may be considered outdoor environments. This standard does not specify design methods but gives input parameters to the design of the building envelope, heating, cooling, ventilation, and lighting, of which the building envelope is also an essential part of the physical built structure in architecture. The building envelope is defined as the physical separator between the conditioned and unconditioned environment of a building, including the resistance to air, water, heat (e.g., Cleveland and Morris, 2009), light, and noise transfer (e.g., Syed, 2012). According to this definition, all spaces starting from (i.e., bordered to) the immediate vicinity of the building facade may be considered to be outdoor spaces. Thus, an outdoor space may be partially protected by walls of a building or sheltered by a cantilever or canopy of a building. An outdoor space may also be under the open sky, for example, on the roof terrace of a building or totally apart from any building. Being under an open sky outdoor space may be partially sheltered or offering protection from weather.

The vicinity of the building brings into discussion the definition of *semi-outdoor environments*. Instead of choosing building envelope as a common nominator, they may be seen in relation to thermal environments, where they fall between the categories of indoor and outdoor environments (Nakano and Tanabe, 2020). An indoor environment provides controlled thermal comfort, whereas in an outdoor environment occupants need to adjust themselves to achieve thermal comfort, clothing adjustment being one of the principal forms of behavioral adaptation (Nikolopoulou and Steemers, 2003; Nakano and Tanabe, 2020). Nakano and Tanabe (2020) emphasize that in semi-outdoor spaces, the degree of environmental control may range from simple shading to moderate air conditioning (open cafes, terraces, arcades, atriums, train stations) where people are likely to expect an environment that is different from indoors. The semi-outdoor environment defined through the thermal environment does not exclude closed spaces providing protection from weather but lacking stable thermal control. Thereby, structures enclosing a semi-outdoor environment may have a solid building envelope, but the qualities of it do not fulfill the required standards to maintain a stable thermal indoor climate.

Beyond the building design framework, the thermal environment is another type of approach to defining the concept of a semi-outdoor environment. However, knowledge of thermal comfort and the means to adapt on it lay grounds for understanding how to facilitate the adaptation by spatial and technical solutions, although not all factors are possible to deduce from the photographs or their captions. Nikolopoulou and Steemers (2003) define thermal adaptation as the gradual decrease of the organism's response to repeated exposure to a stimulus, involving all the actions that make them better suited to survive in such an environment. In the context of thermal comfort, this may involve all the processes which people go through to improve the fit between the environment and their requirements. Although Nakano and Tanabe (2020) refer to Nikolopoulou and Steemers (2003) in their approach to considering the concept of adaptation effective, they reference Brager and de Dear's (1998) thermal adaptation classification including behavioral, physiological, and psychological processes, instead of Nikolopoulou and Steemers's (2003) division to physical, physiological, and psychological processes. Brager and de Dear's (1998) behavioral adaptation includes a personal adjustment of clothing, activity, posture, or selection of environment; however, the interaction with the environment is not indicated implicitly as in Nikolopoulou and Steemers's (2003) physical adaptation divided into reactive and interactive adaptation. Reactive adaptation refers to personal changes, altering one's clothing level, posture or position, or metabolic heat (consumption of hot or cool drinks). In interactive adaptation, people make changes to their environment to improve their comfort conditions (opening window, turning thermostat, opening parasol, etc.; Nikolopoulou and Steemers, 2003). Both forms of adaptation have interesting linkages to some key concepts of work environmental research, such as the role of environmental control and personalisation in supporting worker satisfaction (Vischer, 2007). On the other hand, excessive or unsuccessful attempts of behavioral adaptation can be seen as indicators of environmental stress and an unsupportive work environment (Vischer, 2007). The available seating options implicate the possible reactive adaptation, i.e., the possibility to choose posture and position in the space. The possibility for interactive adaption is often mentioned in captions or in other textual information related to the images.

In the current research, physiological adaptation, unlike other forms of adaptation, in the context of the thermal environment (physiological acclimatization) is not seen as having central importance when extreme environments are not under inspection (Nikolopoulou and Steemers, 2003; Nakano and Tanabe, 2020). However, considering cold weather, the Nordic countries have a long and distinct tradition of second-home tourism where more than half of the population has access to them. Together with the tradition of outdoor recreation (Müller, 2007), people are used to operating year-round outside also in cold seasons. Long-term physiological adaptation might have importance concerning geographic location.

Factors concerning psychological adaptation cannot be unequivocally analyzed from the physical environment alone. However, we considered it to be a valuable piece of background information to advance understanding of the affecting immaterial factors. Nikolopoulou and Steemers (2003) name six important aspects meaningful in psychological adaptation: *naturalness, expectations, experience, time of exposure, perceived control*, and *environmental stimulation*. *Naturalness* indicates that wide changes in the physical environment are tolerated (Griffiths et al., 1987) when all climatic changes occur naturally. *Expectations* about what the environment should be like (instead of being) influence people's perceptions (also Nakano and Tanabe, 2020). *Experience* directly affects people's expectations and can be differentiated in the short and long term, and adaptation levels are established as functions of past exposure (Wohlwill, 1998). Concerning *exposure time*, Nikolopoulou and Steemers (2003) report that exposure to discomfort is not viewed negatively if the individual anticipates that it is short-lived. Generally, unless exposure to discomfort is threatening to the living organism, tolerance to the thermal environment is great. Nikolopoulou and Steemers (2003) assume that sensitivity to the cold is greater than to heat; however, this might also be a matter of the naturalness, experience, and even long-term acclimatization of, for example, native Nordic people. Nikolopoulou and Steemers (2003) continue that *perceived control* plays an important role in tolerating wide variations. They claim that it is widely acknowledged that people, having their own choice to expose themselves to certain conditions, become more tolerant to the thermal environment. They point out that it is increasingly believed that *environmental stimulation* is preferred, whereas a static environment becomes intolerable. According to the present IEQ norms (e.g., BS ISO 17772-1, 2017), the stable conditions are considered as the desired state.

*Microclimate* and *adaptivity* are also intertwined. Walton et al. (2007) have developed a comfort index that measures adaptivity in outdoor spaces. They have reported gustiness and wind speed as being the most important in determining user satisfaction. They emphasize the importance of microclimatic factors for comfort in the outdoor space (Zacharias et al., 2001; Walton et al., 2007). Facilitating people's possibilities to protect themselves from the weather, including exposure to gusts and wind speed, is an essential part of architectural design and the qualities of the built environment in general, Walton et al. (2007) emphasize. A microclimate is not visible in the built environment; however, it is possible to deduce the visible structures that might mitigate the climatic conditions.

### 2.1 Microclimate: about architectural means to comfort in the built environment

In architectural design, the semi-outdoor space is not a defined concept. From the Industrial Revolution onwards, the concept of comfort has been one of the central driving forces of society (Maldonaldo and Cullars, 1991), and its effect can be seen also in the relationship of architectural practice toward climate control. Within this paradigm, exposure to weather is an enemy of economic efficiency and productivity (Roesler, 2022). The IEQ is one application of this ideal, but on an urban scale, it has resulted in what Shove (2009) calls a “fortress-like strategy”—maintaining a standardized bubble of protected indoor space which keeps out variable and “threatening” outdoor weather conditions. Depending on the climatic zone, the reasons for protection are different but have resulted in similar urban forms of indoor spaces, i.e., shopping malls and arcades, interiorised streets, and “interiors on the move”—cars, buses, and trains which carry their passengers between air-conditioned spaces, from home to office to shopping venues and transport interchanges (Chang and Winter, 2015). Although alternative approaches have also coexisted (see, i.e., Requena-Ruiz, 2006; Roesler, 2016), thermal modernity remains the dominant way of perceiving and inhabiting built space (Chang and Winter, 2015). Designing weather-controlled spaces is also a social issue: In affluent areas, more investments are made to create favorable microclimates (Roesler, 2016). In contrast to typical outdoor work, the weather has not traditionally affected knowledge workers. This homogenizing paradigm has caused many other aspects of climate relevant to architecture to be neglected which can be brought to the foreground by examining the relationship between people, climate, and the built form from socio-cultural or anthropological viewpoints. In addition, the demand for more sustainable built environments requires departing from the prevailing energy-intensive paradigm and cultivating approaches that prioritize low-carbon design and center social practice in adapting to the climate (Chang and Winter, 2015).

The architectural design solutions are central in affecting the microclimatic conditions since it is not only the microclimate itself that people perceive but also the surrounding spatial settings. Spatial qualities and space type, such as the appearance of materials and heights of the buildings, change the experience (Eliasson et al., 2007; Lenzholzer, 2010). However, design recommendations on microclimate issues are sparse and scattered. This might be due to the visually inclined tradition in urban design and the fact that the effects on other senses, such as the thermal sense, have not been widely considered (Lenzholzer and van der Wulp, 2010) or the fact that designing for urban microclimate involves dealing with a relatively high degree of complexity (i.e., taking into consideration of the day or season when the place is used; Lenzholzer and Brown, 2016).

Examining semi-outdoor spaces from an architectural design perspective also requires understanding the social factors determining the perception of the space, in addition to the physical dimension. For example, people are often engaged in activities, and those activities might be associated with physical amenities such as outdoor furniture. To understand the functional side of outdoor spaces on a practical level, Gehl (1971/1987) divides urban activities into optional and necessary. Necessary ones are performed despite the outdoor conditions. This relationship between functional use and microclimatic conditions has been confirmed by several studies, showing that weather conditions that are considered comfortable increase the number of people present in an outdoor space (Eliasson et al., 2007). When examined from this kind of functional perspective, climate becomes a background which hinders or favors certain affordances and activities. In other words, climate controls activity. *Climate-sensitive urban design* (Carmona et al., 2003) understands the effects of microclimate on how accessible the (non-climate-related) design affordances are for the users—in the context of this research, this means different work-related activities.

Another proof of the negotiable nature of the concept of comfort is that people enjoy experiencing different weather conditions (Eliasson, 2000). Heschong (1979) calls this celebration of microclimatic diversity a “thermal delight.” In the context of urban design, actively including experiences of climate within the design schemes can be called *climate-revelatory* design. Within a space, this could mean, i.e., movable elements that can be shifted to change microclimatic conditions, which opens up new potential for architectural experiences but also enforces the experience of nature in the city (Lenzholzer, 2012).

Whereas, IEQ is a concept defined by building physics and building service engineering, examining the climatic diversity and taking the abovementioned aspects into account require examining the material aspects of built reality from an architectural and anthropological viewpoint (Roesler, 2016). This can mean embracing a broader definition of comfort examining ethnographically the contemporary activities (such as outdoor office work) and how people achieve comfort through environmental control and personal or social practice. Examining the range of transitory indoor–outdoor boundaries in the public and private sphere and actively cultivating the many ways in which we already interact with the indoor–outdoor conditions is central in creating a new understanding of adaptive comfort strategies (Shove et al., 2008). Therefore, examining knowledge work in outdoor environments contributes to the development of a novel understanding of the reciprocity between people, microclimate, and spatial settings. While doing this, it is central that the research setting acknowledges the material nature of the climatic phenomena instead of succumbing to the primacy of the visual, as is often the case in architecture (Roesler, 2016).

## 3 Outdoor and semi-outdoor environments as knowledge work environments

Although outdoor environments for knowledge work have been only sparsely studied, the prerequisite factors of what constitutes a good work environment indoors (e.g., Vischer, 2007, 2008) are, in our view, a relevant starting point for conceptualizing the outdoor work environment. In Vischer's (2007, 2008) environmental comfort model of workspace quality, the environment supports the productivity and wellbeing of a worker through physical, functional, and psychological comfort. The physical qualities of the environment must meet basic human needs, such as those of safety, accessibility, and hygiene, with a certain minimum threshold to make the occupying of the environment possible. Such factors are covered by building codes and regulations in indoor spaces, while the preconditions of outdoor working are different. The indoor regulations do not apply to designing urban environments favorable for outdoor knowledge work, and such design guidelines have not yet been defined. Functional comfort, in turn, refers to the environmental characteristics that support the performance of work-related tasks, such as appropriate lighting, furniture ergonomics, and suitable arrangements, e.g., concentrative work or collaboration (Vischer, 2007, 2008). The third dimension—psychological comfort—refers to psychological needs related to the work environment, such as feelings of belonging, privacy, ownership, and environmental control (Vischer, 2007, 2008). Applying these concepts to the potential characteristics of outdoor environments, the restorative effects of green environments on human wellbeing (Hartig et al., 2003; Berto, 2014) could also, in our view, be viewed as psychological comfort factors in the outdoor work environment, while attentional restoration and other cognitive effects of nature (Berman et al., 2008; Williams et al., 2018) could also support functional comfort.

A good fit between the person and work environment is related to higher satisfaction and better productivity (Edwards et al., 1998). Hypothetically, the attractiveness and increased use of outdoor environments could be related to such settings providing a higher variety of environmental resources and options, increasing the chances that an individual is able to create a work environment that meets his/her work-related and personal needs. In this study, we focus on the physical aspects of the work environment as functional and mainly psychological dimensions of the environment are more difficult to evaluate from photographs. However, in interdisciplinary multimethod research, the quantified qualitative photo analysis outcomes would be combined with other datasets, e.g., psychological aspects could be part of the study.

Generally, some prerequisites of different outdoor work activities can already be recognized (Mangone et al., 2017; Petersson Troije et al., 2021). The physical, functional, and psychological conditions required for concentrated work (e.g., reading) include sufficient weather protection (e.g., rain, wind, direct sunlight/glare, and temperature), a place comfortable enough to sit down, and protection from the unwished-for feeling of being watched from behind (Petersson Troije et al., 2021). Architectural solutions can facilitate working in outdoor and semi-outdoor environments. Therefore, designers need to understand the features of thermal conditions and aspects of outdoor adaptation to design favorable action possibilities for outdoor knowledge workers.

**Figure 1:**
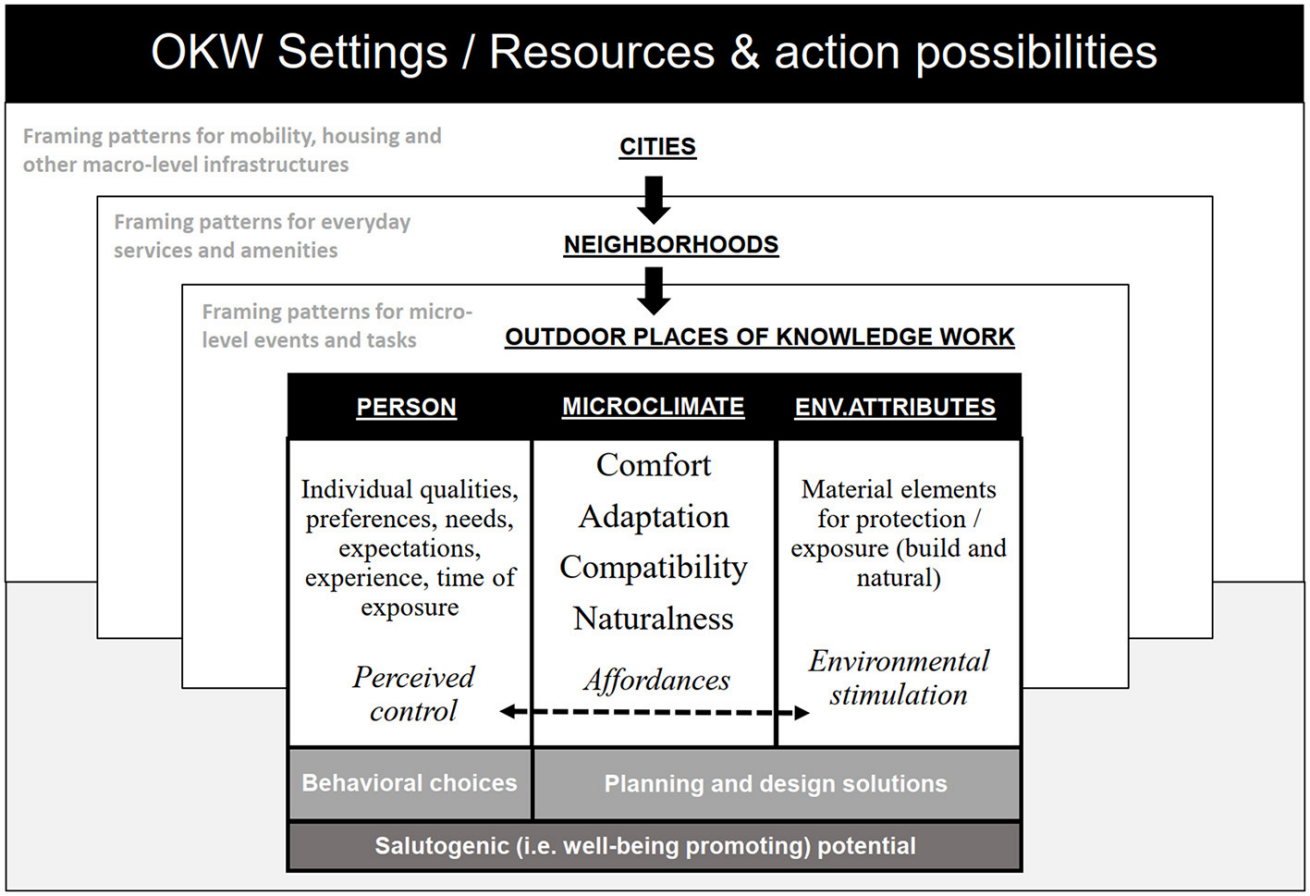
Nested domains of outdoor knowledge work settings.

The built environment is an important determinant of activity patterns, and favorable preconditions that encourage people to spend more time outdoors can be supported by design. According to the theory of affordances (Gibson, 1979), which focuses on the dynamic relation of perception and action, we do not just see the world of objects but rather a world of opportunities for action (Heras-Escribano, 2019). Affordances thus “offer,” “provide,” or “furnish” a range of possibilities for action, either for good or ill, meaning that positive affordances are potentially beneficial to the person, while negative affordances are potentially harmful (Maier and Fadel, 2009). Physical, cultural, and social context-dependency of affordances (Borghi, 2021) means that environmental affordances become perceived and actualised by individuals in certain contexts, when they are relevant and meaningful. This can also be called environmental compatibility, or person-environment fit. According to sociologist Aaron Antonovsky, the mechanisms generating health and wellbeing (salutogenic potential) are firmly linked to a sense of coherence, which is formed through three main components: comprehensibility, manageability, and meaningfulness. The planning and design solutions where environmental attributes are well-considered have the potential to support its comprehensibility to persons. The favorable action possibilities (affordances, such as microclimatic conditions), where the person may choose (manageability) options fitting best to individual qualities, preferences, and needs, are most likely to support a sense of meaningfulness (Figure 1). If the perceived environment is outside the individual's optimal range, an overload of stressors impedes coping. If the coping strategies are successful, adaptation occurs (Antonovsky, 1996).

The affordances of outdoor environments have been previously studied mainly from children's and adolescents' perspectives (Aradi et al., 2015; Kyttä et al., 2018; Sando and Sandseter, 2020). However, from a wider perspective, the salutogenic (i.e., wellbeing promoting) potential of outdoor green spaces has been researched, e.g., in over 60 studies in the form of eight perceived sensory dimensions (PSDs): natural, serene, sheltered, cohesive, open, cultural, social, and diverse (Qiu and Nielsen, 2015; Stoltz and Grahn, 2021; Stoltz, 2022). Each dimension indicates a generally perceived psychological need that requires support in the environment (Grahn and Stigsdotter, 2010; Grahn et al., 2010), thus acting as the experiential qualities of environments that account for their salutogenic effects. According to research, the need and preference for different PSDs in the outdoor environment vary based on the mental and physical state of the individual (Grahn et al., 2010). The more stressed an individual feels, the higher seems the need for the more restorative PSDs (nature, serene, sheltered, cohesive) to be, while the preference for the other PSDs rises simultaneously with the level of the individual's wellbeing and need for more outward directed engagement (Stoltz, 2022). Stoltz and Grahn (2021) emphasize that both green and built environments and features may function as salutogenic affordances in multiple ways and that research in health-promoting environments needs to move beyond this dichotomy of “green” vs. “gray”. According to Stoltz and Grahn (2021), the ongoing research on PSD answers the need to identify in more detail the specific qualities important in order for different environments to support salutogenic processes efficiently. Architects and urban planners have much power in framing the default options for the environmental arrangement, which the users either accept, try to change, or invent alternative ways of utilizing it. Therefore, control over, manageability, and governance of space are important questions related to working environments and wellbeing. Individual creativity and the possibilities of a worker to influence his/her work-related settings and personal needs are linked to letting multiple affordances compete, without providing a predetermined way of solving the competition (see Borghi, 2021).

## 4 Applying journalistic photo analysis to images of outdoor knowledge work

Photo analysis belongs to qualitative research enabling one to increase the overall understanding, in our case, of the quality, characteristics, and meanings of the places people prefer to use as voluntarily chosen outdoor places for knowledge work. Although the number of studies on outdoor knowledge working is low, other researchers have also recognized the potential of photographic methods in this research topic (Florin and Lehtinen-Jacks, 2022). In this study, we develop further the outdoor knowledge work purposes customized PPSA-pOKW model (Herneoja et al., 2022) for interdisciplinary research collaboration when needing to combine datasets. The key approach is to quantify the qualitative visual contents of the photographs in numeric form. This quantification enables both to use of the machine vision in the analysis phase (for large quantities of photographs) and thematizing findings in quantified form. This second version of the model introduced in this study is called for now on the pOKW2 model. Both models, PPSA-pOKW, and the developed one, pOKW2, originate in press photograph analysis methodology (Kedra, 2013). Our interest was motivated by the possibilities we recognized in interdisciplinary research where photo analysis could be used as one of the multimethod approaches. The mobile-based collecting of self-reported photographs by identified occupants provided the possibility to attach the time-location stamp to each photograph. In this study, our focus is on photographs documenting the occupant's view (gaze) from the place of work instead of documenting the outdoor “workstation” itself as in the PPSA-pOKW model. One broadly used mobile-based method is the experience sampling method (ESM) which provides opportunities for collecting rich self-reported and objective data, including photographs, in outdoor knowledge work. The ESM enables the repeated collection of time- and location-specific data about the use, perceptions, and objective qualities of different hybrid working environments naturally used by the participant. The ESM may include multiple choice questions, location data (GPS coordinates), time of responding, and a photograph of the environment taken by the respondent, e.g., the gaze direction when working. The photograph is important because the GPS coordinate does not have direction. The time-location dimension enables time-dependent datasets (e.g., health data measured by wearable electronics, and weather data: wind speed and gust, and temperature) to combine with, e.g., tracking data (GPS coordinates), to explore relations between participants' health measurements and their physical environment. The quantification of the photo analysis outcome would set the basis for combining all the collected data by ESM.

Press Photograph Story Analysis (PPSA) by Kedra (2013) will form the basis of both the PPSA-pOKW model (Herneoja et al., 2022) for photographs documenting the outdoor workplace and the second version of our model (pOKW2) for self-reported photographs documenting the occupant's gaze (to be collected by ESM). We review the similarities and differences of our models in relation to the original PPSA model (Kedra, 2013) and to each other.

**Figure 2:**
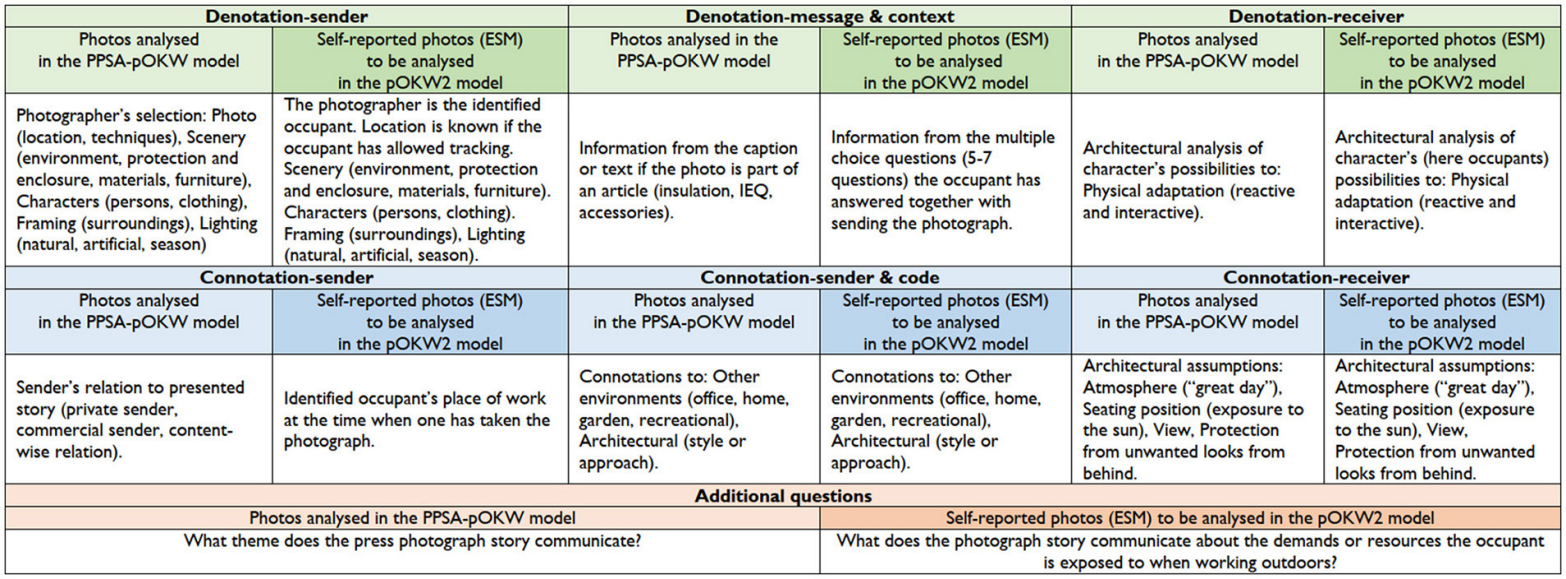
Side-by-side columns indicate the photographs, either analyzed in the PPSA-pOKW model or self-reported ones by the occupant (when answering the sent ESM survey) to be analyzed in the pOKW2 model.

The classification categories for these photo reportage-type journalistic images are content, context, layout, number of photographs, and dominant function (Wolny-Zmorzyński, 2007, 2010; Kedra, 2016). In the original criteria, the content was defined as everyday life situations. However, we focussed on photographs where the content was about places designed or used for outdoor and semi-outdoor knowledge work. The rest of the classification criteria did not need modification from the original (Kedra, 2016). Photo reportage-type journalistic images are considered to be visual communication and, therefore, we applied the Press Photograph Story Analysis (PPSA) by Kedra (2013): sender, message, code, context, contact, and receiver. These components of visual communication formed the basis of dividing the data into thematic sections relating to the PPSA model by Kedra (2013): denotation (denotation-sender, denotation-message and context, denotation-receiver), connotation (connotation-sender, connotation-message and code, connotation-receiver), and additional questions (Figure 2). The thematic sections are divided into two columns: the descriptions used in the PPSA-pOKW model on the left and the descriptions of self-reported photographs (ESM) to be analyzed in the pOKW2 model on the right.

In the PPSA-pOKW model, the photographs were collected from the visual discovery engines to test the model. In these cases, the sender was not only the photographer but might be the person who posted the photograph or the commercial supplier who purchased a specific type of photograph. In the second version pOKW2, the photographs would be self-reported attachments to the ESM surveys, and therefore, the sender would always be the photographer. In our case, the receiver is a professional architect designer and work environment researcher, instead of a person in general. The contact was not included in Kedra's (2013) model PPSA because mass-mediated communication always involves a spatial and social distance between the participants (e.g., McQuail, 1997; Kedra, 2013). In the PPSA-pOKW model, we included contact information of the photograph's location referring to either website address/-s as a virtual location (to find the image later), a keyword as a content-wise contact information and geographic location of the photo's content (if available) to support the interpretation of the thematic grouping. In the pOKW2 model, the participant's (sender's) contact information would be included in the consent, and the exact location would be known because of the tracking data of the ESM survey. In the PPSA-pOKW model, we kept the context as it was in the original model PPSA (Kedra, 2013), focussing on the caption as a central element of the page context in photography reception (e.g., Müller et al., 2012), but also considering texts beyond caption if additional information was provided. In the pOKW2 model, with self-reported photographs, the ESM survey's multi-choice questions would provide additional information about the context. In addition, the time-location-specific information of the photograph would enable in-depth analysis of the context, e.g., specific weather conditions including gust and direction of wind. Kedra (2013) refers to Barthes (1977) to explain code in press photography: Image is not the reality, but at least it is a perfect analogon. In the PPSA model, the denotative part is an analogon. In both versions of our model, the denotative part is looked at with the architect design researcher's expertise combined with the understanding of knowledge work environment researcher and, therefore, the field-specific features are recognized. In Kedra's (2013) PPSA model, the connotative part is a sign that requires an interpretant, the receiver, since the photographic code provides the receiver's intertextual connotations. According to Barrett (2006), we make meaningful connections between what we see and experience in a photograph and what else we have seen and experienced.

The additional question in the original PPSA model (Kedra, 2013) was developed for the learning process purposes but also to provide a summary for the analysis. The additional question was also encouraged to be formulated according to the specific research topic in the PPSA-pOKW model as we did by asking what theme does the press photograph story communicate (of the outdoor or semi-outdoor workplace)? Our aim was also to be able to thematize the findings of the analysis. In the pOKW2 model, the additional question was rephrased to form what theme does the photograph story, supported by time-location-specific data, communicate about the demands or resources the occupant is exposed to when working outdoors?

**Figure 3:**
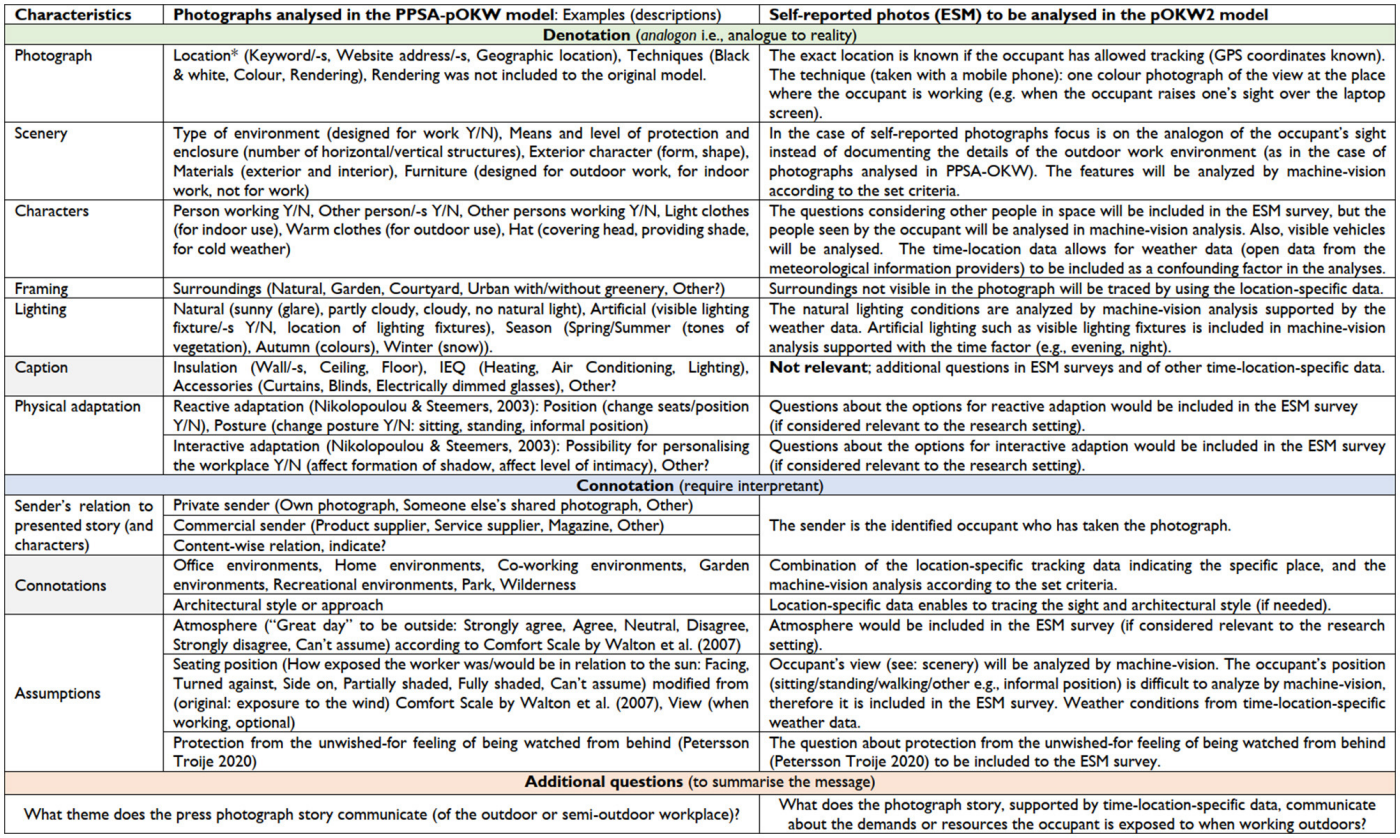
The table details the characteristics and examples (descriptions) of the modified PPSA model outlined in Figure 2. Side-by-side columns indicate either the photographs analyzed in the PPSA-pOKW model or self-reported ones by the occupant (when answering the sent ESM survey) to be analyzed in the pOKW2 model, where the time-location data is included.

The analyzed features structured in Figure 2 were customized according to the content and relevance applicable to the outdoor knowledge work context in Figure 3. The left-hand side column of Figure 3 presents, in detail, the characteristics and examples (descriptions) analyzed from the photographs of the PPSA-pOKW model columns of Figure 2. In the PPSA-pOKW model (Herneoja et al., 2022), the main limitations were the multitude of characteristics (together with their descriptions) and not considering the large quantities of photographs to be analyzed. The right-hand side column of Figure 3 presents the pOKW2 model of Figure 2, describing how the multimethod approach, utilizing other time-location-specific datasets, enables reducing the number of characteristics analyzed from photo reportage images. This development paves the path for handling large quantities of photographs to quantify the outcomes of the thematic analysis. In this second version of our model, pOKW2, the machine vision analysis would provide a crucial aid for the photo analysis process. To that end, we would use state-of-the art approaches, such as deep learning techniques, to analyse the images and their content. Modern publicly available software tools (e.g., TensorFlow, PyTorch, and Keras) and public image libraries to train classifiers for our purposes could be used to identify contextual metadata about the photographs. These data include such as the activities being performed in the photos, detecting specific objects within the images, or, e.g., if the image was taken outdoors, indoors, or in other specific locations. The detailed procedure for the machine vision analysis will be reported in another forthcoming study.

**Figure 4:**
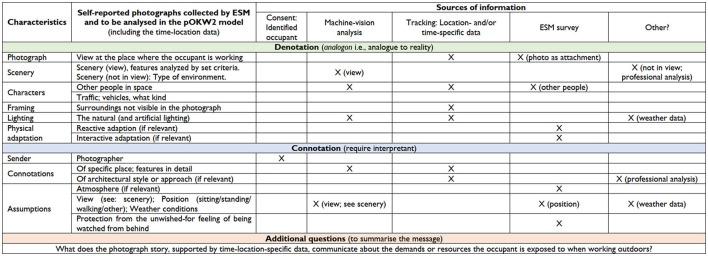
The table presents sources of information to be used in the pOKW2 model to combine/merge data sets with machine-vision analysis outcomes to answer the Additional question.

**Figure 5:**
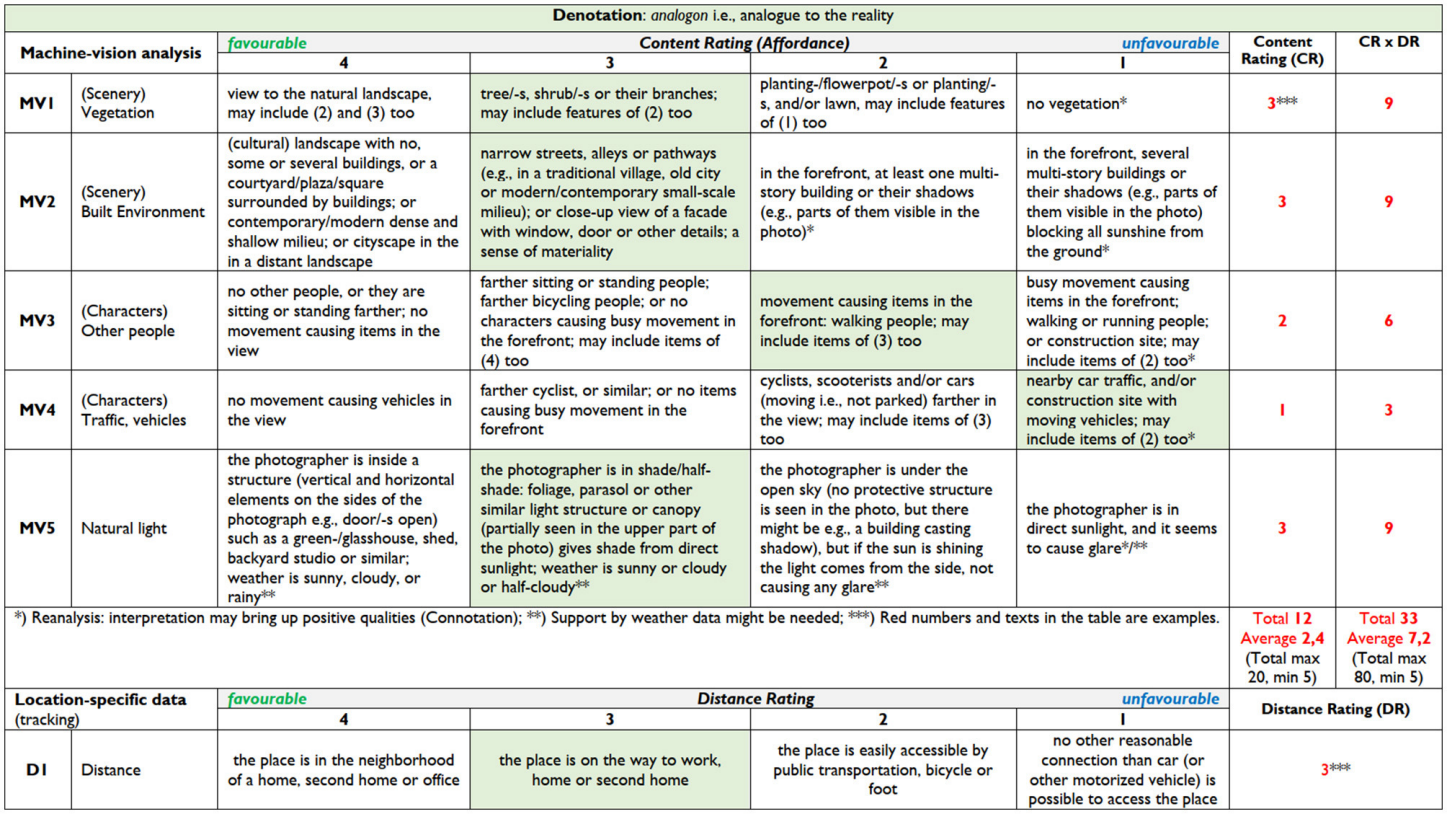
This is the first attempt to rate analog content of reality (Denotation) in the numeric form, first for the machine-vision analysis and later for combining with other datasets. In this first trial, the distance of the place (where the photograph is taken) is given a rating (DR) too to indicate/study how probable it would be that a person would use the same place again for outdoor work. In this DR, also, physical activity-supporting means are valued.

**Figure 6:**
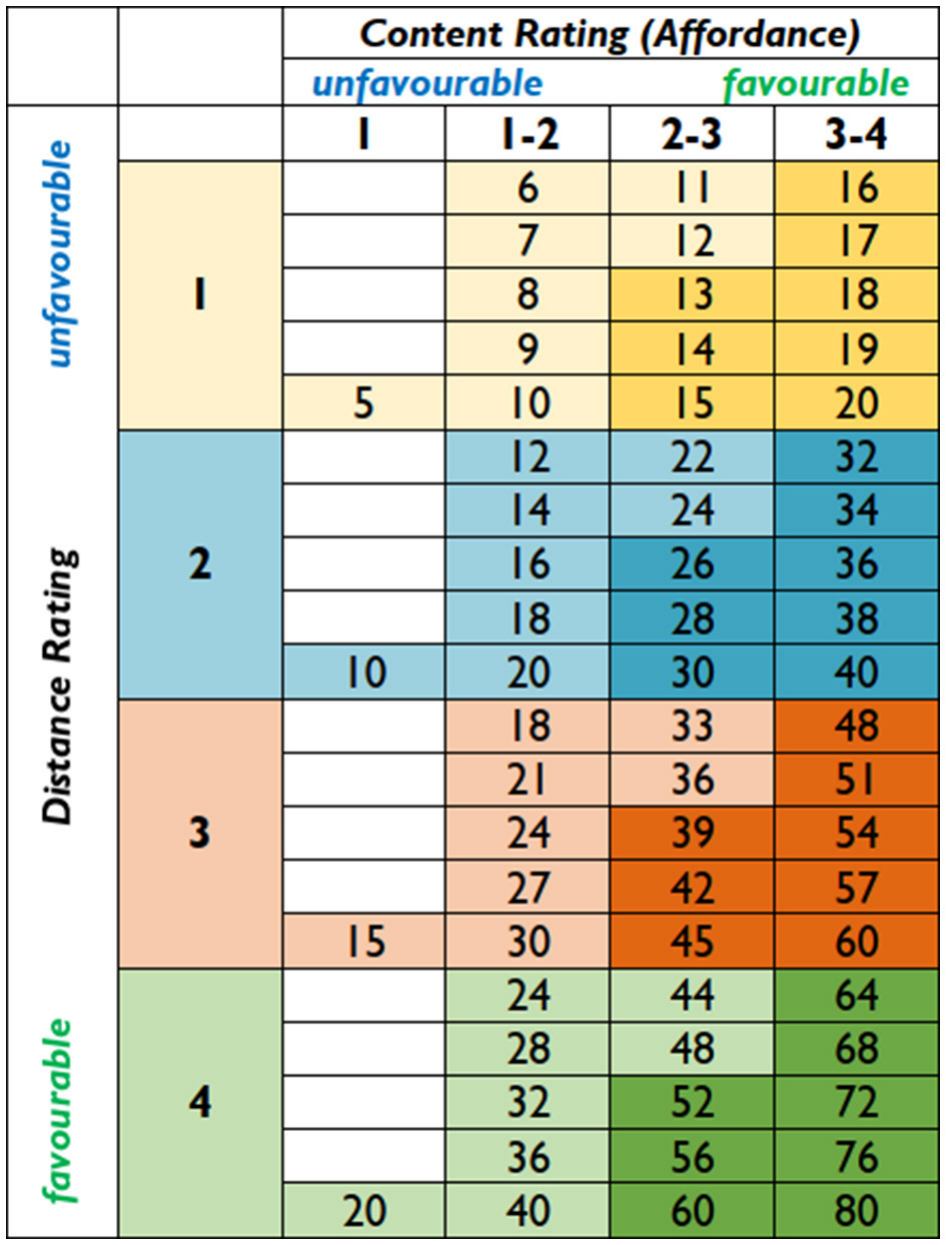
Table shows in numeric form the possible outcomes of Content rating (CR) multiplied by Distance rating (DR). The highlighted numbers indicate Content rating average of 2,6/5 or higher, before multiplying it with DR.

In Figure 4, we have gathered different sources of information potentially available when using the ESM survey method. In this study, we focus on developing a numeric rating for the qualitative content of the photograph for machine vision analysis purposes (Figure 5). In forthcoming interdisciplinary research projects, we will combine machine vision analysis outcomes with other datasets to understand better the demands and resources the occupant is exposed to when working outdoors. Furthermore, those projects will contribute open datasets to further solidify the usefulness of the method as machine vision-based tools will require accurate training data. In the example (Figure 5), the distance of the photo spot is given a rating (DR) depending on how far it is from home, second home, or office to indicate how probable it would be that a person would use the same place again for outdoor work. In this DR, physical activity-supporting means are also valued together with sustainability. The table (Figure 6) shows in numeric form the possible outcomes of content rating (CR) multiplied by distance rating (DR), highlighting with a darker tone the upper half of the CR average (2,6/5 or higher before multiplying it with DR). In addition to the location-specific DR in the forthcoming research projects, it is interesting to combine it with time-location-specific weather data (temperature, speed, and gustiness of wind), not to mention receiving additional information from the ESM survey. The time-specific device-based health data would provide evidence of the effects of outdoor working in specific places.

The limitation of the second version of our model is that the photograph does not document the actual workplace but only the view representing the gaze of the occupant. Of course, we could ask the occupant to take another photograph to document the actual “outdoor workstation” and send it with the documented view as an attachments when answering the ESM survey. We hesitate to ask for two photographs as it can be challenging to give instructions for photography, which may lead to failure in data collection. If we take the risk to include also the “outdoor workstation” photograph, we could exploit the knowledge gained from the PPSA-pOKW model (Herneoja et al., 2022) and develop it suitable for machine vision analysis, as in the second version of our model.

## 5 Remarks on the test study of the PPSA-pOKW model

In parallel with gathering the material for the test study, we were able to test and improve the PPSA-pOKW model (Herneoja et al., 2022) further. Data were collected from Pinterest's English pages, and about 50 of them were tested with the PPSA-pOKW model. The subject areas of the photographs were mainly from the UK or northern continental Europe. The photographs were collected by using keywords referring to outdoor work. It was noteworthy that there was not an established wording to name places for outdoor knowledge work. We started the search with the keyword *outdoor office*. The images represented as places of outdoor knowledge were divided into five types: Pods, Sheds, Studios, Sunrooms, and Greenhouses. The complete list of used keywords (in italics) to collect photographs on Pinterest is attached to each subtitle describing the typology. The posted photographs often included the original website where the photograph was found and called with other terms. These synonyms enabled us to extend the search by also using other keywords. The photographs were placed in an online canvas tool Miro to enable arranging the photographs and to make the preliminary thematic grouping by their exterior character easier. The first remarks of the test study are presented here briefly.

### 5.1 Pods

An outdoor *Pod*, also called *Outdoor Office Pod, Outdoor Office Phone Booth, Small Office Pod, MicroPod*, or *Garden Work Hub*.

In general, these Pods form a similar group (character and looks) as the Pods in the indoor work environments, except *Garden Work Hubs* resembling regular garden sheds, furnished to serve as workplaces. In indoor offices, in solo use, Pods are meant to offer places for work tasks requiring concentration, preventing workers from acoustic or visual distractions, or protecting others from the disturbing noise the solo worker inside the Pod is creating (e.g., video meetings and phone calls). Outdoors is less probable that the solo worker would cause any disturbance to someone else, but, instead, one needs sufficient protection from the weather. However, the outdoor Pods do not take advantage of the transitional zone between the interior and exterior space around it.

### 5.2 Sheds

An outdoor *Shed*, called *Shed Home Office, Backyard Office Shed*, or *She-Shed*, was typically an existing garden shed or summerhouse that was taken into use for knowledge work. In windowless garden sheds, the only natural light would enter the interior by keeping the door open. The summerhouses usually had many windows; thereby, the glare would be more of a challenge than the lack of natural light. The interior and furnishing were more home or garden-like than the Pod interiors' office atmosphere. Some of these shed photographs were from suppliers' catalogs; however, also many of them were seemingly modified by the private people themselves.

### 5.3 Studios

One group of outdoor workplaces are the unique *Backyard Studio* being designed by architects for a specific client. These Studios are small in size, usually including a desk for one or two people. Their architecture is ambitious although the used materials are simple and inexpensive. The given reasons for the building project were the need for more room because of a growing family, running a home office, and willingness to stay in the area where housing prices are known to be high.

### 5.4 Sunrooms

*Sunroom Offices* form a group of outdoor workplaces indicating how existing semi-outdoor spaces in detached houses or townhouses are re-occupied as spaces for office work. These glass-metal structured spaces demonstrate how rooms not having fully insulated building envelopes and lacking continuous stable IEQ are still used as, at least, temporary workplaces.

### 5.5 Greenhouses

Converted *Greenhouse Office* represented one thematic group where the existing building type was utilized for the purposes of outdoor knowledge work, instead of growing plants. The greenhouse provided a wind shelter with plenty of light. In the photographs, greenhouses were placed in a half-shadow created by tree foliage. The challenge seemed to be still to prevent glare and balance with the thermal climate.

We were aware of the limitations of the data analyzed with the PPSA-pOKW model in this test study. The photographs were from a social media context, and the complex internal logic of algorithms using artificial intelligence and machine learning caused an uncontrollable factor (Liu et al., 2017), together with the possible commercial interests (Lo et al., 2016). Still, with these known limitations, we were willing to analyse the found images represented as places of outdoor knowledge (Herneoja et al., 2022). In addition to this incoherent data quality, we recognized the limitations in the PPSA-pOKW model (Herneoja et al., 2022). The model had a multitude of characteristics and their written descriptions, and therefore it is laborious, if not impossible, to use for analyzing large quantities of photographs. We realized the analysis model needed simplification for broader use, which was considered in developing the pOKW2 model.

## 6 Conclusion

The environments of multilocational knowledge work tend to be indoors. The context of working outdoors is an emerging and little-studied phenomenon, especially in the context of the built environment. Working outdoors has become technologically and socially possible in multilocational knowledge work. Hybrid work combining on-site working at the workplace and teleworking has increased the spatial and temporal flexibility of working. This complexity of physical places and transitions between them appears as an ecosystem of indoor and outdoor spaces and transitions between these locations. We consider it necessary to understand the developmental needs in different areas of design from micro-level outdoor workstations to macro-level urban structures. In this context of the built environment, the definition of outdoor and semi-outdoor needed clarification in general and as the basis for the photo analysis of the places of outdoor knowledge work.

In our view, outdoors and semi-outdoors may also mean an enclosed and protected space, and not only open sky environments without protection from the weather. The building envelope separates the conditioned indoor environment from the unconditioned outdoor environment. In the built environment, an outdoor space may be partially protected by walls or other parts of the building, and it may also be under the open sky or totally apart from any building. Being under an open sky outdoor space may be sheltered with light structures or provide the occupants with other types of protection from the weather. In outdoor spaces, the thermal adaptation is largely the occupant's responsibility adjusted mainly with clothing adaptation. In semi-outdoor spaces, the degree of environmental control is broader, varying from simple shading to moderate air conditioning where people are likely to expect an environment differing from indoors. The thermal environment of semi-outdoor spaces does not exclude enclosed spaces providing protection from the weather but lacking stable thermal control as indoors. From now on, we consider both outdoor and semi-outdoor environments as possible places of outdoor knowledge work.

Thermal control in semi-outdoor environments is related to strategies on how people adapt to the surrounding thermal climate. Especially interesting was the structuring of the physical adaptation of a reactive and interactive adaptation. The reactive adaptation included personal changes, altering one's clothing level and posture or position, which the latter ones can facilitate with design solutions. The interactive adaptation comprises the changes people make to their environment to improve the comfort conditions, which have, interestingly, a linkage to the ideas of control and personalisation—key concepts from work environmental psychology—supporting worker's sense of environmental satisfaction. In addition, physiological and psychological adaptation provides valuable background knowledge for architectural design researchers about occupants' relation to being in outdoor or semi-outdoor environments.

In a built environment, according to the theory of affordances, the dynamic relation of perception and opportunities for action can be supported by design. Environmental affordances become perceived and actualised by individuals in certain contexts, when they are relevant and meaningful, such as in the case of environmental compatibility or person-environment fit. The mechanisms generating health and wellbeing are linked to a perceived sense of coherence, either as being outside the optimal range impeding coping or adaption occurs if coping strategies are successful. The present development of emphasizing that green and built environments and features may function as salutogenic affordances in multiple ways lays favorable grounds for interdisciplinary interactions. In the built environment, the microclimatic conditions together with the surrounding spatial settings, such as the appearance of materials and heights of the buildings, change the experience.

We had identified interest in places of outdoor office work in popular media such as the visual discovery engine Pinterest and started exploring the places of outdoor knowledge work through photo analysis to understand their manifestations further. In this study, we took a step further and simplified the photo analysis to better fit interdisciplinary research on wellbeing and health, where large quantities of self-reported photographs from identified occupants would be analyzed. Future studies could apply this method, for example, together with the mobile-based experience sampling method (ESM) where photograph/-s with time-location-stamp and short survey data would provide additional information. The use of ESM would make it possible to simplify the photo analysis phase to pave the path to machine vision analysis. For example, the time-location-specific multimethod approach would provide the option to use other datasets as sources of data beyond the photographs alone.

The Press Photograph Story Analysis (PPSA) by Kedra (2013) provided a solid ground for analysis of the visual discovery engines' photographs and the possible self-reported photographs attached to the ESM survey. The difference between the contents was that in the PPSA-pOKW model (Herneoja et al., 2022), the photograph documents the actual workplace, but in the second version of our model (pOKW2), the photograph would be a document of the view representing the gaze of the occupant. Of the original PPSA model, only the *message* of the six elements: *sender, message, code, context, contact*, and *receiver*, remained the same in our models.

In the PPSA-pOKW model (Herneoja et al., 2022), the *sender* was not only the photographer but might also be the person who posted the photograph or the commercial supplier, but in the second version, the photographs would be self-reported with the ESM survey and therefore the *sender* would always be the photographer. The *receiver* was considered in both versions of our model an architect design researcher and a work environment researcher. Unlike in the original PPSA model, the contact information was included: the photograph's location, a keyword as a content-wise contact, and the geographic location of the photo's content to support thematic grouping. In the second version of our model, the participant's (sender's) *contact* information would be known because of the tracking data of the ESM survey. In the PPSA-pOKW model (Herneoja et al., 2022), we kept the *context* as it was in the original model, focussing on the caption, but in the second version, the ESM survey would provide additional information about the *context* of the self-reported photograph. In addition, the time-location-specific information of the photograph would enable in-depth analysis of the *context*, e.g., specific weather conditions.

With *code*, the model was structured into two: *denotation* being analog to the reality shown in the photograph and *connotation* requiring an interpretant, the receiver, supporting the systematic analysis considering both image types in separate columns. In the original PPSA model, the third element was the *additional question* to provide a summary for the analysis and to thematize the findings. In the original PPSA model, the third element was the *additional question* (AQ) to provide a summary for the analysis and to thematize the findings. We developed the PPSA-pOKW Herneoja et al. (2022)'s AQ: *What theme does the press photograph story communicate (of the outdoor or semi-outdoor workplace)?* In the second version of our model to an AQ: *What does the photograph story, supported by time-location-specific data, communicate about the demands or resources the occupant is exposed to when working outdoors?*

The test study, using the PPSA-pOKW model (Herneoja et al., 2022), seemed to provide a comprehensive tool to produce a rich descriptive analysis of the found photographs, however, being too long and complex to present in the article and not applicable for large quantities of photographs. In this second version of our model pOKW2 with self-reported photographs, we could benefit from time-location-specific datasets by reducing the number of characteristics analyzed. This development would support handling large quantities of photographs and quantifying the thematic analysis with machine vision analysis, for example, when combining datasets in interdisciplinary research cooperation. The limitation of the second version of our model pOKW2 lies in the contents; in other words, the photograph does not document the actual workplace but only the view representing the gaze of the occupant. We hesitate to ask for two photographs, which may lead to failure in data collection. If the “outdoor workstation” photograph would also be required, we could exploit the PPSA-pOKW (Herneoja et al., 2022) of the model to develop it for machine vision analysis, as in the second version of our model.

## Data availability statement

The raw data supporting the conclusions of this article will be made available by the authors, without undue reservation.

## Author contributions

The original idea of defining an outdoor environment as a place of outdoor knowledge work was AHa's and AHe's. The original idea of the customized photo-analysis (PPSA-pOKW) and its development to the second version pOKW2 was AHe's. AHe wrote the first draft of the manuscript with AHa, SM-A, ER, EO, and SH. AHa oversaw the work environment psychology contents of the manuscript. ER, SM-A, EO, AHa, and AHe oversaw the concept of affordance in the urban design context (ER and EO) and outdoor environments (SM-A, AHa, and AHe). EO wrote the Section Microclimate: about architectural means to comfort in the built environment. SH oversaw the technological contents of the ESM and the machine-vision analysis. ER created Figure 1, and AHe created the other Figures 2–6, commented on and approved by all authors. All authors contributed to the manuscript and read and approved the submitted version.
